# Fructose-rich diet leads to reduced aerobic capacity and to liver injury in rats

**DOI:** 10.1186/1476-511X-11-78

**Published:** 2012-06-19

**Authors:** José Diego Botezelli, Lucieli Teresa Cambri, Ana Carolina Ghezzi, Rodrigo Augusto Dalia, Fabrício Azevedo Voltarelli, Maria Alice Rostom de Mello

**Affiliations:** 1 Department of Physical Education, São Paulo State University - UNESP, Av: 24-A, 1515 Bela Vista., 13506-900, Rio Claro, São Paulo, Brazil; 2 Department of Physical Education, Federal University of Mato Grosso, Grosso, MT, Brazil

**Keywords:** Fructose, Liver injury, Oxidative stress, Rats

## Abstract

The main purpose of this research was to investigate the alterations in the aerobic capacity and appearance of metabolic alterations in Wistar rats fed on fructose-rich diet. We separated twenty-eight rats into two groups according to diet: a control group (C) (balanced diet) and a fructose-rich diet group (F). The animals were fed these diets for 60 d (d 120 to 180). We performed insulin, glucose as well as a minimum lactate test, at d 120 and 180. At the end of the experiment, sixteen animals were euthanized, and the following main variables were analysed: aerobic capacity, the serum aspartate aminotransferase (AST) to alanine aminotransferase (ALT) ratio, serum and liver triglyceride concentrations, serum and liver thiobarbituric acid reactive substance (TBARS) concentrations, serum and liver catalase and superoxide dismutase (SOD) activity and haematoxylin-eosin histology (HE) in hepatocytes. The remaining twelve animals were submitted to an analysis of their hepatic lipogenic rate. The animals fed a fructose-rich diet exhibited a reduction in aerobic capacity, glucose tolerance and insulin sensitivity and increased concentrations of triglycerides and TBARS in the liver. Catalase and SOD activities were reduced in the livers of the fructose-fed animals. In addition, the serum AST/ALT ratio was higher than that of the C group, which indicates hepatic damage, and the damage was confirmed by histology. In conclusion, the fructose-rich diet caused significant liver damage and a reduction in insulin sensitivity in the animals, which could lead to deleterious metabolic effects.

## Background

Nonalcoholic fatty liver disease presents like a new marker of metabolic syndrome [1]. This hepatic disease is most frequently diagnosed in the United States, affecting almost thirty million people [2,3]. The aggressive form of this disease is most commonly found in adults, but the number of children affected is growing rapidly [4]. Interest in the causes and consequences of lipid infiltration in the liver has risen in recent years because of the association between triglyceride accumulation in different tissues and the development of insulin resistance [5].

The influx of triglycerides into hepatocytes leads to an overproduction of reactive oxygen species by beta-oxidation, which causes an anti-oxidant/oxidant imbalance [6]. The elevation of pro-oxidant species causes membrane and DNA damage and the inactivation of some regulatory proteins, which causes tissue inflammation and induces insulin resistance, apoptosis, cellular mutations and other effects.

Rats fed a triglyceride-rich diet have been used as an experimental model of human metabolic syndrome. Previous studies have had some success with inducing nonalcoholic fatty liver disease by feeding animals different concentrations of fructose [7,8].

Moreover, physical activity is an important tool for the prevention of metabolic syndrome [8]. It has been shown that physical activity improves glucose tolerance and reduces insulin resistance [8,9]. Aerobic capacity can be a good indicator for physical conditioning and can be used after training or diet interventions to show alterations on physical conditioning parameters.

## Objective

The main purpose of this research was to investigate the alterations in the aerobic capacity and appearance of metabolic alterations in Wistar rats fed on fructose rich diet. This study focused on the appearance of NAFLD and the subsequent alterations as systemic oxidative stress, liver damage, insulin resistance and hyperinsulinemic state.

## Methods

### Animals and treatments

Twenty-eight adult (aged 120 d) Wistar rats were used. The animals were kept in collective cages (four animals per group) at a controlled temperature of 25 ± 1 °C and under a light/dark cycle of 12/12 h with free access to water and food. The experiment was performed at the Nutrition, Metabolism and Exercise Laboratory at São Paulo State University, Rio Claro, Brazil. The weights of the animals were recorded weekly during the experimental phase, and the area under the curve (AUC) was calculated with Microsoft Excel 2007 software using the trapezoidal method [10]. All experiments were analysed and approved by the Biosciences Institute Animal Ethics Committee, Rio Claro Campus (case number: 005/2010).

### Experimental groups

At 120 d of age, the animals were separated randomly into two groups: the Control Group (C) was fed a commercial balanced diet from 120 to 180 d of age, and the Fructose Group (F) was fed a semi-purified, fructose-rich diet from 120 to 180 d of age.

### Diet composition

We used commercial chow (Labina, Purina®) as a control diet (57,3 % carbohydrate, 41,2 % of cornstach). For the fructose-rich diet, we used an adapted diet standardised by Bezerra and colleagues (2004) composed of (in g/kg) 202 of casein, 625.5 of fructose, 2 of l-cysteine, 70 of soy oil, 35 of mineral salt mix (AIN-93MX) [11], 10 of a vitamin mix (AIN-93MX)[11], 50 of fibre and 2.5 of choline chloridrate.

### In vivo assays

#### Oral glucose tolerance test (oGTT)

The oGTT were performed at 8^th^ week of experiment after the animals had fasted for 12 h. The first blood sample was collected by a small tail punction to determine the basal glucose and insulin concentrations (t0). Next, a 20 % glucose solution (2 g/kg of animal weight) was administered via an intragastric catheter, and samples for glucose and insulin were collected 30, 60 and 120 min later into four heparinised capillaries calibrated to 25 μl each. Glucose levels were analysed by the glucose- oxidase method, and insulin levels were assessed using the radioimmunoassay method [12]. The results were analysed by calculating the glucose and insulin AUC using the trapezoidal method [10] in the Excel 2007 software.

#### Insulin sensitivity (ITT)

Insulin sensitivity was evaluated using the insulin tolerance test at 8^th^ week of experiment. The test consisted of a bolus injection of insulin (300 mU/kg body weight) followed by blood sample collections (to measure glucose concentrations) from a punction at the tip of the tail before and 30, 60 and 120 min after the insulin injection. The serum glucose disappearance rate (Kitt) was calculated using the formula ${0.93/t}_{1/2}$, wherein t_1/2_ is the half-life of the process. The serum glucose half-life was calculated from the slope of a least-square analysis of serum glucose concentrations 0–60 min after the subcutaneous injection of insulin; during this time, the glucose level reduces linearly [13].

#### Minimum lactate test (ML)

Aerobic capacity was analyzed at the 8^th^ week of experiment via the lactate threshold during swimming and calculated by determining the “minimum lactate test” [14], adapted to rats [15]. For this test, the animals were initially placed individually in tanks (100 cm X 80 cm X 80 cm) containing water at 31 ± 1 °C. Animals carried an overload that was 13 % of their body weight to induce hyperlactatemia and were then exercised for 30 sec. After resting for 30 sec, they swam carrying the 13 % load until exhaustion. After a 9 min rest, a blood sample was collected by means of a cut in the distal end of the tail to determine lactate concentration. Animals then performed exercise with progressively heavier loads [15]. The initial load was 2 % of the body weight of the animal; the load was increased 0.5 % every 5 min until exhaustion. After each load change, a blood sample was collected to measure lactate. The lactate minimum swimming workload (ML) was determined using a second-order polynomial curve adjusted to the blood lactate vs. workload curve. The blood lactate concentration was measured by spectrophotometry [16]. The lowest lactate concentration on the curve (minimum lactate) theoretically represents the maximum exercise intensity, where lactate production and removal occur in the same proportions [17].

### In Vitro assays

#### Biological samples

Forty-eight h after the last “in vivo” evaluation (180 days old), sixteen animals (eight per group) were killed under anaesthesia (sodium thiopental, 40 mg/kg of body weight, intraperitoneally) for the collection of two blood samples from the hepatic vein. The first sample was used to obtain serum to evaluate glucose, triglyceride, HDL cholesterol, LDL cholesterol and total cholesterol concentrations as well as the activities of the hepatic transaminases aspartate aminotransferase (AST) and alanine aminotransferase (ALT) enzymes using colorimetric methods (commercial kits LABORLAB®) according to the methods of Nogueira [18]. The second sample was used to determine the TBARS concentration and catalase (CAT) and superoxide dismutase (SOD, commercial kit Cayman®) activities. The liver was removed, and a sample was taken to determine the triglycerides concentration [19]; another sample was used to evaluate biomarkers of oxidative stress (TBARS, catalase and SOD). An additional sample was used for histological analysis using the haematoxylin/eosin method. The twelve remaining animals were used to determine the rate of liver lipogenesis (six per group) [19], also, a sample of the visceral adipose tissue (mesenteric and retroperitonial) was removed to determine triglycerides concentration (calculated as mean of both regions) [18].

#### Biomarkers of the antioxidant defense system

##### Catalase (CAT)

To determine catalase activity, liver samples (100–150 mg) were placed in Eppendorf tubes® containing 1 ml of cooled phosphate buffer, subjected to agitation and centrifuged at 10,000 rpm for 5 min. Catalase activity dosage assays were performed, using phosphate buffer (50 mM) and oxygen peroxide (H_2_O_2_, 10 mM) [20]. The linear reduction in the H_2_O_2_ absorbance values was assessed by spectrophotometry (240 nm) according to the reaction below.

We calculated catalase activity using the formula $\left(2.3/\Delta t\right)*\left(a/b\right)*\left(\log A_{1}/A_{2}\right)$, in which a is the haemolysed volume in the sample cell, b is the total volume of the sample cell, A_1_ is the absorbance value at t = 0 s, and A_2_ is the absorbance 15 s later (t = 15 s) [20].

### Superoxide dismutase (SOD)

Superoxide dismutase activity was determined after washing the samples with PBS (pH 7·4) containing heparin 0·16 mg/L to remove blood cells. Then, the tissue was homogenised (on ice) using 1 ml of HEPES buffer (20 mM, pH 7·2) containing 1 mM EGTA, 210 mM manitol and 70 mM sucrose. Following centrifugation at 10,000 rpm for 15 min at 4 °C, the samples were maintained at −20 °C until the level of activity of total SOD (cytoplasmic and mitochondrial) was determined. For this procedure, we used a commercial kit (Cayman®), and a tetrazolium salt was used to detect superoxide radicals produced by xanthine-oxidase and hypoxanthine. In this reaction, one unit of SOD is defined as the quantity of enzyme necessary to affect 50 % of superoxide radical dismutation [21].

#### Biomarkers of Lipid-c Peroxidation

##### TBARs Concentration

We detected substances that react to thiobarbituric acid (TBARS). This method involves analysing the final products produced by lipid-c peroxidation (lipid peroxides, malonaldehyde and other low-weight aldehydes), which react with 2-thiobarbituric acid to produce Schiff bases. Blood samples were collected, and serum proteins were isolated using phosphotungstic acid. The supernatant was used in a colorimetric reaction with thiobarbituric acid, which uses MDA (the main product of lipid peroxidation), as the standard, and was analysed by spectrophotometry (5 nm). The liver samples (100–150 mg) were placed in plastic tubes containing phosphate buffer and were analysed by spectrophotometry (5 nm) [22].

#### Lipogenic rate in the liver

The lipogenic rate in the liver was measured using tritiated water as a radioisotope marker. Tritium is incorporated into stable C-H bonds during fatty acid synthesis by the exchange of ^3^ H with pyrimidine-reduced nucleotides (NADPH), which are produced via pentose or malic enzymes. The tritiated water was administered via an intraperitoneal injection. After 60 min, the animals were killed, and a liver samples was isolated and the lipids were extracted using the method of Folch et al. [19] and were quantified using liquid scintillation spectroscopy (TRI-CARB 2100TR). The rate of fatty acid synthesis was calculated according to Windmuller and Spaeth [23]:

(1) $$\text{Fatty acid}\;\mu \text{mol}/2\;\text{h}=\frac{\text{dpm of}\;3\text{H}/\text{atom}-\text{g of H}\times \text{t}2-\text{t}1}{\text{dpm of}\;3\text{H incorporated into liver FA}\times 109\times 1}$$

#### Liver haematoxylin-eosin (HE) histology

m. The slices were subjected to the haematoxylin-eosin staining method. The slices were hydrated and stained with haematoxylin (10 min) and were then washed and stained with eosin (5 min). Finally, they were washed and preserved in Canadian balsam [24]. Liver samples were collected and fixed in Bouin’s fixative. The tissue was mounted in HitoResina (Leica) and sliced in a microtome (Leica RM2145) to a thickness of 6

### Statistics

The Shapiro-Wilk W test was used to verify the normality of the sample. The results were analysed statistically using a Student’s t-test.

## Results

**Figure 1:**
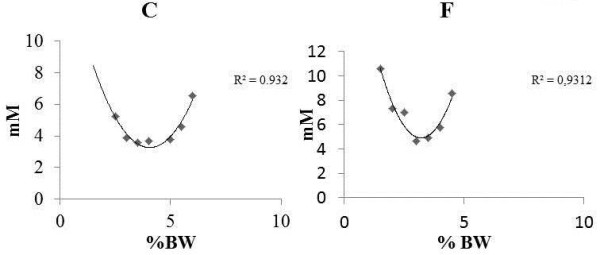
**Minimum lactate test of one animal of each group, as an example.** In these particular cases, the estimated ML was 4.21 % of body weight, while the interpolated blood lactate concentration was 3.96 mM for the C (Control) animal and for F (Fructose) animal we found 2.53 % of body weight and 3.94 mM blood lactate interpolated concentration.

The minimal lactate test is showed in Figure 1. Two rats were used as example to show the lactate changes through the test.

**Figure 2:**
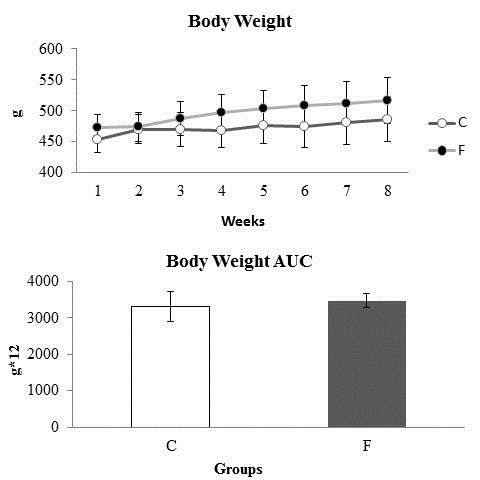
**Body weight change, the area under the curve (AUC) for body weight and the weight gain of the animals during the 8 weeks of the experiment. C:** Control; **F:** Fructose. n = 14 animals per group. *Significantly different from the control group (p ≤ 0·05).

The change in body weight, the AUC of the body weight measurements and of the animals during the 8 weeks of the experiment were not different between the groups (Figure 2).

**Figure 3:**
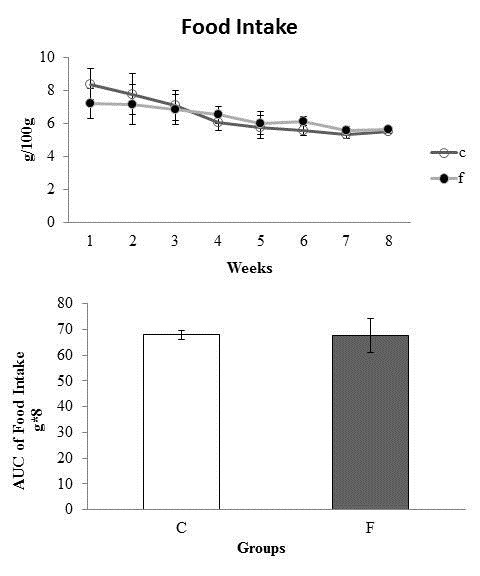
Weekly food intake (g/100 g) and area under the curve for food intake (g/100 g*8). C: Control; **F:** Fructose. n = 14 animals per group. *Significantly different from the control group (p ≤ 0·05).

Similarly, no difference was observed between the two groups, both in weekly food intake and the AUC of food intake (Figure 3).

Table 1 shows the blood lactate concentrations and the workloads corresponding to the minimum lactate for both groups. Fructose feeding reduced the ML without alteration in blood lactate concentrations.

**Table 1:** Blood lactate concentrations (mM) and the workloads (%BW) corresponding to the minimum lactate for both groups

**Groups**	**Rest**	**Minimum Lactate**	
	**mM**	**mM**	**%BW**
**c**	1.3 ± 0.31	3.2 ± 1.2	3.9±0.5
F	1.5 ± 0.2	4.3 ± 0.7	2.5 ± 0.3^*^

C: Control; F: Fructose. n = 8 animals per group.

*Significantly different from the control group (p ≤ 0·05).

Table 2 shows the serum glucose kinetics of the animals during the insulin tolerance test and the Kitt values (glucose disappearance rate) at the end of experiment. The F animals exhibited lower Kitt scores compared with C animals, which indicates insulin resistance.

**Table 2:** Glucose kinetics (mg/dl) and glucose removal rate (%/min, Kitt) during the insulin tolerance test (ITT)

**Serum Glucose and KITT levels in the ITT**					
**Group**	**Time**				**Kitt**
	**0**	**30**	**60**	**120**	
C	85.8 ± 4.5	80.6 ± 5.7	80.5 ± 5.4	91.3 ± 9.3	1.04 ± 0.3
F	87.5 ± 7.4	86.9 ± 5.2	88.3 ± 14.8	95.2 ± 9.0	0.54 ± 0.16*

C: Control; F: Fructose. n = 8 animals per group.

*Significantly different from the control group (p ≤ 0·05).

The serum glucose and serum insulin levels as well as the area under the curve scores for serum glucose and insulin during the oral glucose tolerance test at the end of the experiment are shown in Table 3. The animals fed the fructose-rich diet exhibited higher serum glucose AUC values compared to C. The serum concentrations of insulin and the insulin AUC values were higher in group F compared when to group C.

**Table 3:** Serum glucose kinetics (mg/dl), serum insulin (ng/dl), area under the curve for serum glucose (mg*120 min/dl, AUC) and serum insulin (ng*120 min/dl, AUC) during the oral glucose tolerance test (oGTT)

**Group**	**Serum glucose and insulin in the oGTT**					**Area Under the Curve**
	**Time**					
	**Variable**	**0**	**30**	**60**	**120**	
C	Glucose(mg/dl)	69.6 ± 2.2	80.8 ± 6.7	81.8 ± 4.2	73.4 ± 1.3	9353.7 ± 456.6
	Insulin(ng/ml)	2.1 ± 0.3	2.4 ± 0.1	2.3 ± 0.4	2.1 ± 0.3	277.4 ± 32.6
F	Glucose(mg/dl)	75.2 ± 4.8	90.6 ± 7.3	85.8 ± 3.8	83.7 ± 2.3	10219.9 ± 530.2*
	Insulin(ng/ml)	2.5 ± 0.3^*^	2.5 ± 0.3	3.6 ± 0.5^*^	3.4 ± 0.4^*^	381.2 ± 49.5*

C: Control; F: Fructose. n = 8 animals per group.

*Significantly different from the control group (p ≤ 0·05).

The concentrations of serum glucose, triglycerides, total cholesterol, HDL cholesterol and LDL cholesterol and measurements of liver injury markers (AST/ALT ratio), catalase and SOD activities and lipid peroxidation markers (TBARS) at the end of the experiment are shown in Table 4. The animals fed the fructose-rich diet exhibited higher levels of glucose and triglycerides, an increased AST/ALT ratio, TBARS levels and catalase activity.

**Table 4:** Glucose, triglycerides, total cholesterol, HDL cholesterol and LDL cholesterol concentrations, AST/ALT ratio, SOD activity, catalase activity and TBARS concentrations in animal sera at the end of the experiment

**Variable**	**C**	**F**
Glucose (mg/dl)	83.6 ± 4.5	98.5 ± 10.5*
Triglycerides (mg/dl)	123.4 ± 49.0	268.3 ± 35.1*
Total cholesterol (mg/dl)	91.7 ± 9.6	96.3 ± 15.5
HDL cholesterol (mg/dl)	42.7 ± 5.3	43.5 ± 6.9
LDL cholesterol (mg/dl)	62.8 ± 3.9	65.4 ± 1.3
AST/ALT ratio	1.08 ± 0.4	2.8 ± 0.8*
SOD (U/ml)	1.7 ± 0.4	1.5 ± 0.6
Catalase (U/ml)	42.2 ± 10.4	68.4 ± 6.8*
TBARS (μM)	16.5 ± 2.0	22.2 ± 4.4*

C, Control; F, Fructose. n = 8 animals per group.

*Significantly different from the control group (p ≤ 0·05).

As shown in Table 5, fructose-fed group exhibited higher concentrations of triglycerides and TBARS in the liver than the control group. Conversely, fructose-fed animals showed a reduction in liver catalase activity.

**Table 5:** Triglyceride concentrations, SOD activity, catalase activity and TBARS concentrations in animal livers at the end of the experiment

**Variable**	**C**	**F**
Triglycerides (mmol/mg)	6.4 ± 1.9	72.8 ± 25.7*
SOD (umol/min.mg protein)	4.69 ± 1.6	2.6 ± 1.4
Catalase (umol/min.mg protein)	5.7 ± 1.8	2.2 ± 1.1*
TBARS (umol/mg)	0.30 ± 0.03	0.35 ± 0.02*

C, Control; F, Fructose. n = 8 animals per group.

*Significantly different from the control group (p ≤ 0·05).

**Figure 4:**
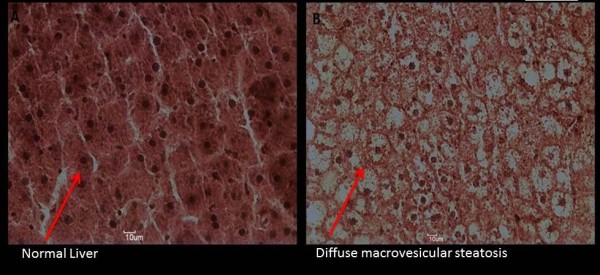
**Liver HE histology of one rat at the end of the experiment as a representative example. A:** Control Group; **B:** Fructose Group.

The HE histology is presented in Figure 4. The image shows a substantial increase in fatty accumulation in hepatocytes (diffuse macrovesicular steatosis) in the animals fed the fructose-rich diet.

**Figure 5:**
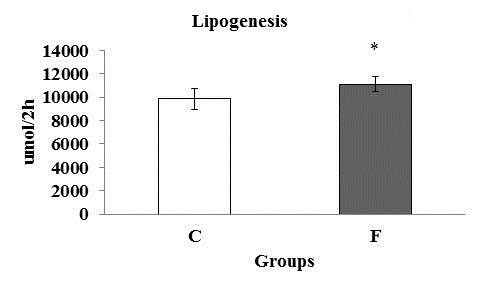
**Lipogenesis rate (μmol/2 h) of six rats at the end of the experiment. C:** Control; **F:** Fructose. n = 6 animals per group. *Significantly different from the control group (p ≤ 0·05).

Figure 5 shows the values of lipogenic rates. The animals fed the fructose rich diet revealed an increase in lipogenic rate.

**Figure 6:**
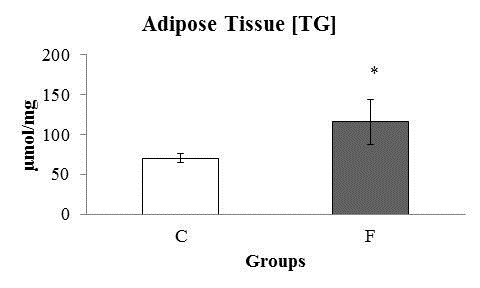
**Triglycerides concentration (μmol.mg) in the visceral adipose tissue of eight rats at the end of the experiment. C:** Control; **F:** Fructose. n = 8 animals per group. *Significantly different from the control group (p ≤ 0·05).534.

Figure 6 shows the triglycerides concentration in the visceral adipose tissue (retroperitoneal and mesenteric region) of the animals at the final of experiment. F showed high concentrations of triglycerides compared to C group.

## Discussion

Previous studies have shown that an increase in metabolic syndrome incidence is highly correlated with changes in alimentary behaviour. Western diets, which are rich in sugars and saturated fats, have been implicated as the main cause of the global pandemic in obesity, glucose intolerance, type-2 diabetes mellitus, hypertension and other conditions [25,26]. Once our main purpose is to investigate changes in the aerobic capacity and the main metabolic alterations caused by the fructose-rich diet administration, we performed an eight weeks experiment starting 120 d of age.

At d 180, the animals in this group had similar weights to those fed a normal diet. This finding corroborates other studies that showed that animals fed on fructose diet for a short term did not present excessive weight [8,27,28].

The exercise is an important and powerful weapon against the metabolic syndrome markers. The minimal lactate zone is situated in a transition domain between intense and severe exercise. In this zone, both glycolytic and oxidative pathways contribute to the energy production, and a reduction on the efficiency in one of them can be responsible for lower performance. In this sense, we performed this test to evaluate the aerobic capacity of the animals. The lactate response allowed us to determine the minimal lactate intensity in both groups and revealed that the aerobic capacity of F group was reduced if compared to C after 60 days of fructose-rich diet consumption. Associated with the reduced aerobic capacity, the F group presented insulin resistance.

Several studies have reported an association of changes in aerobic capacity, insulin resistance and obesity [29-31], but the cause of these alterations was not completely elucidated. Some studies suggest that the insulin can modulate the production and activity of glycolytic and oxidative enzymes [32,33]; thus an insulin resistance could reduce the mitochondrial efficiency and, at least in part, can be responsible for the reduced workloads at the same lactate concentrations showed in the F group. In a previous study our group showed that, rats fed on fructose-rich diet had higher lactate concentrations in a same overload during the maximal lactate steady state test [34].

This insulin resistance occurrence in F group leads to both overproduction and secretion of insulin by the beta cells, then triggering the hyperinsulinemic state, as found during the oral glucose tolerance test. Over 60 d, the F group exhibited a rise in the serum glucose AUC value and a pronounced elevation in both serum insulin concentrations and serum insulin AUC in this test. This hyperinsulinemic state can strongly increase the insulin resistance in peripheral tissues and different organs [35], which reduce, again, the glucose uptake and, consequently, lead to a considerable increase in glycaemia, as was observed in F group, characterizing a vicious cycle. The impairment on carbohydrates metabolism is compensated with higher contributions of lipids as the mainly source of energy.

The fructose metabolism occurs in the liver, which has a great capacity to uptake and phosphorylates this nutrient. This nutrient can be transformed in glucose and glycogen, but this pathway is very “inefficient”. So, the liver choice is to produce pyruvate, which is transferred to mitochondria and is transformed in fatty acids. These fatty acids are used as the mainly liver energy source, stored as triglycerides depots or released in the blood stream as VLDL and NEFA. This characteristic makes fructose a highly lipogenic nutrient [36]. In the present study, the F animals showed higher concentrations of triglycerides in the serum, liver and visceral adipose tissue; in addition, higher liver lipogenic rate was found. This result corroborates several studies that demonstrated the lipogenic capacity of fructose [36,37]. The excessive production and uptake of triglycerides can be viewed in the Figure 6. The animals fed on fructose rich diet presented bigger triglycerides content. High concentrations of liver triglycerides lead to an increase of the activity of fatty-acyl-coA oxidase activity and also stimulate the liver to produce energy trough beta-oxidation. The fructose-rich diet used in our study induced an elevation in liver TBARS concentrations and a reduction in hepatic catalase activity. The higher levels of blood TBARS concentrations in the F group can be correlated with an overutilization of non-esterified fatty acid (NEFA) as energy source. Also, NEFA can induce insulin secretion by pancreatic islets, leading to a hypersulimenic state, as presented by the F group. High insulin levels can downregulate the Malonil-CoA activity, reducing the mitochondrial NEFA transport via CPT-1, leading to an excessive NEFA oxidation at peroxisomes and endoplasmic reticulum [38-41]. Fatty oxidation in these cytoplasmic organelles areas releases high amounts of reactive oxygen species, which can damage mitochondrial membranes (rich in polyunsaturated fat), producing lipid peroxidation metabolites. These metabolites are extremely toxic to the cells and are released into the bloodstream, as observed in the F group [42]. These events could cause an acute systemic response that leads to changes in the antioxidant system, denoted by the higher levels of serum catalase enzyme observed in the present study, which represent a protective mechanism in order to reduce the cellular damage in the F animals [43]. Interestingly, Kakkar et al. [44] demonstrated that Sprague–Dawley rats showed an increase in anti-oxidant activity for several weeks followed by a dramatic reduction in the anti-oxidant response, which consequently increased the level of cellular damage. The chronic imbalance of reactive oxygen species production can impair the ability of the anti-oxidant system to reduce the levels of these radicals, which attenuates its protective function [43].

Figure 6 Minimum lactate test of one animal of each group, as an example, In this particular case, the estimated ML was 4.21 % of body weight, while the interpolated blood lactate concentration was 3.96 mM for the C animal and 2.53 % of body weight and 3.94 mM blood lactate interpolated concentration.

The imbalance between the production and the removal of free radicals is intimately linked to a structural damage and cellular injury; this was confirmed by the increase in the AST (aspartate aminotransferase)/ALT (alanine aminotransferase) ratio (markers of liver damage) in the F animals. Alterations in the levels of serum AST and ALT are considered important markers of hepatic injury and liver fibrosis [45].

Stored hepatic triglycerides are related to acute local insulin resistance. This mechanism can be resulted of a local pro-inflammatory response which alters the circulating AST/ALT ratio [8]. This response releases high amounts of pro-inflammatory mediators such TNF-α and IL-6, reducing the hepatic insulin sensitivity. These substances impair the ability of insulin receptor substrate 1 (IRS-1) and insulin receptor substrate (IRS-2) to be phosphorylated in tyrosine as well as stop the insulin signal and all processes related to glucose uptake [46-49]. Moreover, high levels of serum NEFA leads to insulin overproduction and secretion by the pancreatic beta-cell.

This sequence of events results in a massive influx of triglycerides into beta cells and increases the energy production through the beta-oxidation, resulting in this way, in a cellular lipotoxicity, which can generate structural damage and deficient beta-cell regulation [49]. The animals’ glucose disappearance rates after exogenous insulin administration (Kitt) values were reduced drastically in the F animals, indicating reduction in the insulin sensitivity [35]. This reduction could be responsible for the reduced aerobic capacity, increase on fatty triglycerides, overproduction of EROS; the last may induce liver and islet apoptosis in a long term [25,48,50-52]. Moreover, these findings are closely connected to the appearance of type-2 diabetes mellitus in NAFLD and hypertriglyceridemia patients [1].

## Conclusions

In summary, the fructose-rich diet administration protocol impaired aerobic capacity, induced fatty liver disease and trigger the hypertriglyceridemia, hyperglycaemia, insulin resistance and glucose intolerance, all markers of metabolic syndrome. Furthermore, the fructose reduced the antioxidant activity, enhanced the EROS production and damaged the liver of the animals. These findings may provide the foundation for future studies to elucidate the role of fructose in metabolic syndrome and to develop new treatment procedures for related disorders.

## Competing interests

The authors declare that they have no competing interests.

## Authors' contributions

JDB was responsible for the experimental design, data collection, statistical analysis and preparation of the manuscript. LTC, ACG and RAD were responsible for the data collection and the preparation of the manuscript. FAV was responsible for the preparation of the manuscript. MARM was responsible for the experimental design, financial support, and coordination of the research and preparation of the manuscript. All authors read and approved the final mauscript.
